# Novel endoscopic cryoprobe extraction of acute food bolus impaction: a case report

**DOI:** 10.1016/j.igie.2025.07.006

**Published:** 2025-07-11

**Authors:** Michaela Henderson, Nicole Du, Lori A. Zimmerman, Jessica Yasuda, Denis Chang, Gary Visner, Peter D. Ngo

**Affiliations:** 1 Department of Pediatrics, Boston Children's Hospital and Harvard Medical School, Boston, Massachusetts, USA; 2 Department of Pediatrics, Boston Medical Center and Boston University School of Medicine, Boston, Massachusetts, USA; 3 Division of Gastroenterology, Hepatology and Nutrition, Boston Children's Hospital and Harvard Medical School, Boston, Massachusetts, USA; 4 Division of Pulmonary Medicine, Boston Children's Hospital and Harvard Medical School, Boston, Massachusetts, USA

## Abstract

Acute food bolus impaction is frequently encountered in pediatric patients with underlying esophageal conditions. Traditional endoscopic methods for removing friable boluses, such as meat, are often time-intensive and challenging. This report explores the novel application of cryoadhesion, or freezing and adhering an object to a cryoprobe, for esophageal meat bolus extraction. A 25-year-old man with trisomy 21 and eosinophilic esophagitis presented with acute food bolus impaction. After unsuccessful conventional endoscopic maneuvers, a cryoprobe was externally attached to an endoscope and used to achieve cryoadhesion and extraction of the meat bolus. Within 10 minutes, the meat bolus was successfully removed attached to the cryoprobe without mucosal injury. The patient tolerated the procedure well and was discharged the next day without adverse events. Cryoadhesion via a flexible cryoprobe may offer a feasible alternative for removing challenging food impactions. Further investigation is warranted to evaluate its safety and efficacy.

## Introduction

Acute food bolus impaction is a common reason for urgent endoscopy in children and is even more frequently seen in older adults. Pediatric and young adult patients who develop acute food bolus impaction tend to have predisposing conditions such as eosinophilic esophagitis, dysmotility, or repaired esophageal atresia.^1^, ^2^, ^3^ Numerous cases detail the difficult and time-consuming nature of endoscopic removal of food boluses, particularly those of friable consistencies such as meat.^4^, ^5^, ^6^ Proposed solutions for removal have included using suction techniques, a coagulation probe to burn an anchor hole in the bolus, or a wire-guided balloon for removal.^5^, ^6^, ^7^

This case details the novel use of a flexible cryoprobe to remove an esophageal meat bolus endoscopically via the process of cryoadhesion, or freezing and adhering an object to the tip of a cryoprobe. Gastrointestinal (GI) cryotherapy has used either a spray catheter or cryoablation balloon to ablate target tissue.^8^ However, a through-the-scope cryoprobe has been used during bronchoscopy to obtain biopsy specimens and remove foreign bodies in the airway, particularly organic matter.^9^^,^^10^ Despite their potential GI use, existing cryoprobes are not available in lengths that fit through reusable flexible GI endoscopes with a working channel that is typically 120 cm in length. To our knowledge, this case is the first GI use of a cryoprobe and the first use of cryoadhesion for esophageal food bolus removal.

## Case description

A 25-year-old man with trisomy 21, eosinophilic esophagitis with proximal and distal esophageal narrowing, and previous food impaction of pork meat requiring esophagogastroduodenoscopy with prolonged, piecemeal extraction presented with sudden-onset coughing, dysphagia, and inability to tolerate saliva that began while eating beef stew in the evening. There was no relevant family or social history. On examination, the patient was overall comfortable appearing with a soft abdomen and stable vitals and was observed to be spitting into a cup. Esophagram showed distal esophageal lumen obstruction consistent with food impaction.

The next morning, with the patient under general endotracheal anesthesia, multiple endoscopic tools were used, but because of the friability of the meat and resistance at the mild upper esophageal stricture (15-mm inner diameter), only small pieces of meat could be removed. Tools attempted included a grasping device (Raptor; STERIS, Mentor, Ohio, USA), 4-cm retrieval net (STERIS), distal scope cap attachment (Olympus, Tokyo, Japan), and tripod grasper (PolyGrab FG-600U; Olympus). In addition, an attempt to advance the bolus into the stomach with a 13-mm dilation balloon (Merit Medical, South Jordan, Utah, USA) and grasping device was unsuccessful.

**Figure 1:**
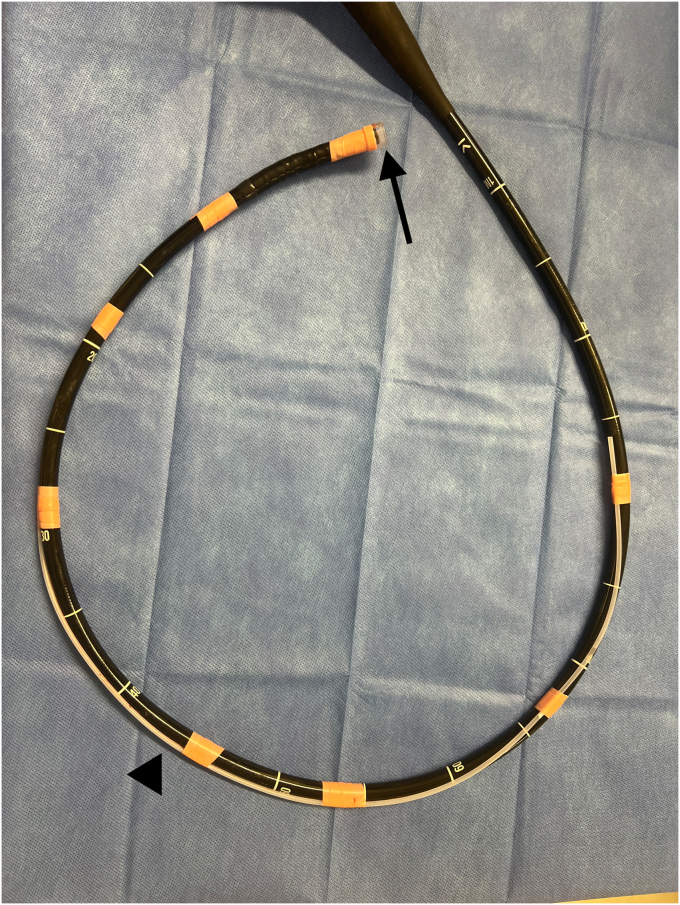
Attached external sheath (*black arrowhead*) and endoscope cap (*black arrow*) to accommodate cryoprobe use.

**Figure 2:**
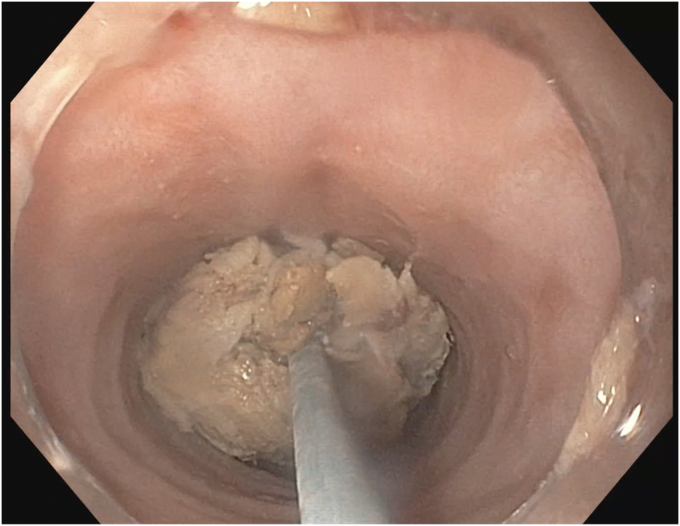
Cryoprobe activation in the center of the food bolus.

**Figure 3:**
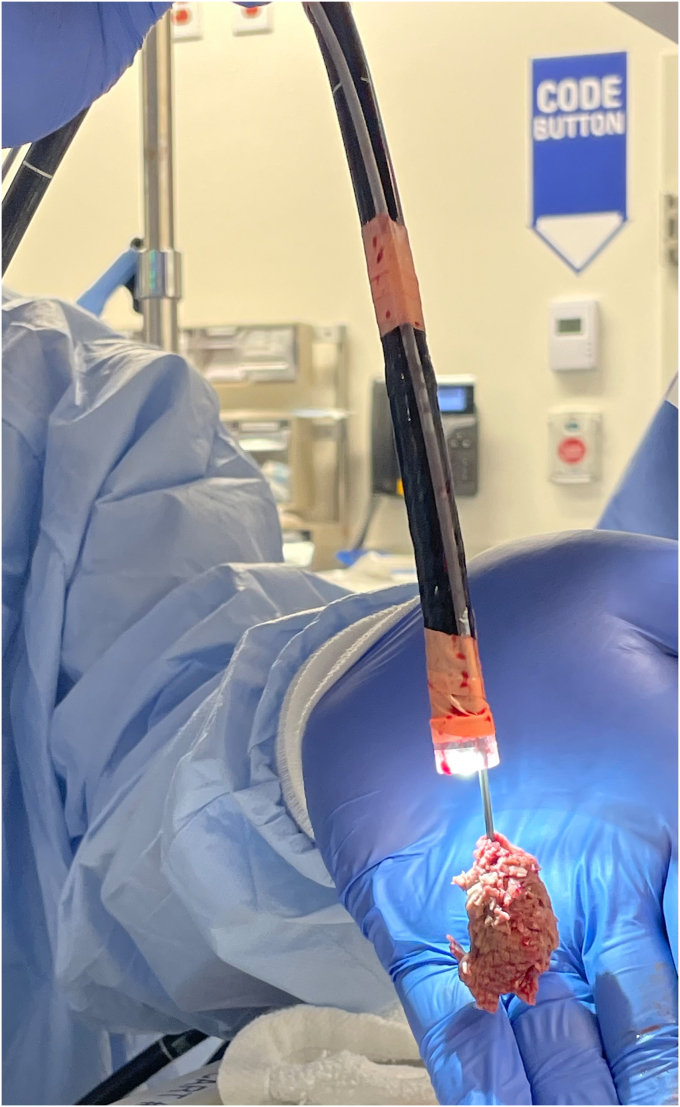
Food bolus attached to cryoprobe advanced through sheath taped to outside of gastroscope.

After 120 minutes of unsuccessful endoscopic maneuvers, guardian consent was obtained for use of a cryoprobe to remove the meat bolus. An 80-cm cut portion of catheter sheath from a 4-cm retrieval net with the inner wire and net removed (Roth Net Platinum-Universal; STERIS) was passed under a 13.4-mm distal scope attachment (Olympus) and secured to the side of a GIF-H190 endoscope (Olympus) every 10 to 15 cm with surgical tape (Fig. 1). We inserted the scope to the level of the meat bolus, and the 1.7-mm outer diameter cryoprobe (Erbe, Tübingen, Germany) was advanced through the sheath under direct visualization into the center of the bolus, taking care to avoid contact with the esophageal wall (Fig. 2). Two attempts with the cryoprobe were taken to remove the bolus within 10 minutes. On the first attempt, the cryoprobe was activated on the cryoextract setting, effect 1, for 15 seconds, which successfully achieved cryoadhesion of the meat to the probe, but upon removal with continuous probe activation, the meat bolus met resistance at the proximal esophageal narrowing and upper esophageal sphincter, dislodging the bolus, and only a 3-mm frozen piece was removed. On the second attempt, the probe was first activated within the bolus for 15 seconds, the probe with attached bolus was briefly moved up and down the esophagus to ensure no cryoadhesion of the bolus to the esophagus, and the probe was then activated for an additional 15 seconds. The probe, sheath, and scope were then removed with continuous probe activation over a duration of 10 seconds, and the meat bolus was removed attached to the probe (Fig. 3). The bolus measured 4 cm × 2 cm. The esophagus was then visualized with both distal and proximal mild benign stenoses and rings but without any signs of cryogenic injury.

The patient tolerated the procedure well, was advanced to soft foods that evening, and was discharged home after an overnight observation, and the patient and family were pleased with the outcome. No adverse events were reported during or after this procedure.

## Discussion

Large food boluses of friable textures often require difficult and time-intensive endoscopic removals, and prolonged procedure time can increase risks of aspiration and perforation.^4^^,^^5^ We demonstrate the feasibility and efficacy of using a cryoprobe for cryoadhesion and removal of an impacted food bolus.

Cryoadhesion was used to both solidify a friable food bolus to avoid piecemeal extraction and to adhere the bolus to the cryoprobe, using the probe itself as a tool for bolus removal. In our approach, it was necessary to insert the cryoprobe into an external sheath attached to the scope, as the 100-cm-long probe is too short to advance through the endoscope channel. New, shorter 80-cm single-use endoscopes with a 2-mm working channel (EvoEndo; Centennial, Colo, USA), or alternatively the manufacture of longer cryoprobes, may allow for through-the-scope GI cryoprobe use. It is possible that excessive probe activation within a food bolus could cause cryoadhesion of the frozen food bolus to the esophageal wall and cause cryogenic injury. Therefore, during the removal, it was important to start with a shorter activation time and increase incrementally. In addition, moving the bolus while still within the lumen before removal through a sphincter or narrowing can ensure cryoadhesion to the bolus without bolus adhesion to the esophageal wall. Although cryoadhesion to a cryoprobe was effective in this case, this work is limited by the single-case report nature, and additional study is needed to confirm the safety and efficacy of this technique.

This successful use of cryoadhesion demonstrates the promise of flexible cryoprobes in food bolus removal as well as their potential to shorten procedural time in difficult cases. The failure of conventional removal devices and the need to modify existing endoscopic tools to accommodate the use of a cryoprobe in this case highlights a need for a broader range of endoscopic equipment and devices.

## Declaration of generative Artificial Intelligence and Artificial Intelligence–assisted technologies in the writing process

During the preparation of this work, the authors used ChatGPT (OpenAI, San Francisco, Calif, USA) in order to generate an initial abstract. After using this tool/service, the authors reviewed and edited the content as needed and take full responsibility for the content of the publication.

## PATIENT CONSENT

The patient in this article has given written informed consent to publication of their case details. This report was determined to be Institutional Review Board-exempt.

## Disclosure

All authors disclosed no financial relationships.
